# Return to work and its determinants among head and neck cancer survivors: a cross-sectional study

**DOI:** 10.4317/medoral.27525

**Published:** 2025-10-14

**Authors:** Ivan José Correia-Neto, Lucas Lacerda de Souza, Leticia Beatriz da Cruz Santos, Juliana Vianna Pereira, José Guilherme Vartanian, Luiz Paulo Kowalski, Pablo Agustin Vargas, Alan Roger Santos-Silva, Fábio de Abreu Alves, Márcio Ajudarte Lopes

**Affiliations:** 1 Oral Diagnosis Department, Piracicaba Dental School, University of Campinas (FOP-UNICAMP), Piracicaba, São Paulo, Brazil; 2 Department Of Stomatology, A.C. Camargo Cancer Center, São Paulo, Brazil; 3 School of Dentistry Federal University of Amazonas (UFAM), Manaus, Amazonas, Brazil; 4 Department of Head and Neck Surgery and Otorhinolaryngology, A.C. Camargo Cancer Center, São Paulo, Brazil; 5 Head and Neck Surgery Department and LIM 28, University of São Paulo Medical School, São Paulo, Brazil

## Abstract

**Background:**

Return to work is an important marker of rehabilitation. While many head and neck cancer survivors are motivated to resume work, they often face greater disability and reintegration challenges than other cancer survivors due to clinical and psychosocial factors. The objective of this study was to identify determinants of return to work and to explore barriers and facilitators to workforce reintegration among head and neck cancer survivors.

**Material and Methods:**

This cross-sectional study of 215 head and neck cancer survivors at two stomatology services used questionnaires to assess socioeconomic, clinical, illness-related, employment, and psychosocial factors. The statistical analysis evaluated correlations with return to work.

**Results:**

Among 215 participants, 137 (63.7%) returned to work. Smaller tumors (p &lt; 0.0001), no lymph node (p = 0.0076), or distant metastases (p = 0.0122) positively influenced reintegration. Radiotherapy (p = 0.0005) and chemotherapy (p = 0.0001) negatively impacted. Completing treatment within six months improved reintegration (67.5%; p = 0.0004), while tracheostomy reduced it (12.5%; p = 0.0021). Neck hypersensitivity (p = 0.0009), paresthesia with incomplete eye closure (p = 0.0027), paresthesia of the corner of the mouth (p = 0.0098) and functional restrictions hindered reintegration. White-collar workers (75.0%) returned more often. Returning survivors had higher anxiety (69.3%; p = 0.0366), quality of life (89.1%; p &lt; 0.0001), higher income (p = 0.0022), and education (p = 0.0110).

**Conclusions:**

Socioeconomic status, education level, tumor characteristics, and treatment modalities significantly affect return to work rates. Physical and functional impairments, along with differences in occupational categories, further hinder reintegration, underscoring the importance of tailored strategies.

## Introduction

Head and neck cancer (HNC) is a critical global health issue, affecting regions such as the skin, lip, oral cavity, nasal cavity, paranasal sinuses, salivary glands, larynx, and pharynx, including the nasopharynx, oropharynx, and hypopharynx (1 , 2). It is the seventh most common cancer worldwide, with approximately 930,000 new cases and 450,000 deaths reported annually (3). Major risk factors include tobacco use, alcohol consumption, and human papillomavirus (HPV) infection. Although HNC has traditionally affected men over 50 years of age, recent years have seen an increase in cases among younger individuals, signaling a shift in the demographic profile of this disease (1 , 2 , 4 - 6). Despite improvements in early detection and multimodal treatments leading to higher survival rates (6 - 9), many HNC survivors face significant challenges in returning to work (RW), which can disrupt their daily routines, social connections, self-esteem, and financial stability (7 , 8 , 10 , 11).

For survivors, particularly those of working age, RW represents a reintegration to society (11 , 12). However, compared to survivors of other cancers, such as breast cancer, HNC survivors often face unique and complex barriers to RW, including a higher risk of disability (1). Many of these difficulties stem from treatment sequelae such as trismus, dysphagia, dysgeusia, xerostomia, osteoradionecrosis, speech difficulties, reduced physical fitness, weight loss, changes in appearance, and psychological distress (1).

Studies on RW among cancer survivors remain focused predominantly on other cancers, leaving a scarcity of data specific to HNC survivors (12). These discrepancies are particularly evident in Brazil, where only one research has explored the employment experiences of HNC survivors (13). As these individuals live longer with the effects of treatment, the financial, functional, and psychosocial challenges they face have far-reaching implications, not only for their quality of life but also for society and employers, given the substantial productivity losses associated with their condition (1 , 11).

This study aims to evaluate the employment status and RW among HNC survivors in a Brazilian cohort. It investigates how socioeconomic conditions, clinical and disease-related factors, treatment side effects, and psychosocial aspects influence work reintegration. By examining these variables, we aim to better understand post-treatment employment dynamics and identify the factors that hinder successful reintegration into the workforce.

## Material and Methods

Ethics statement

This study was approved by the Ethics Committee of the Piracicaba Dental School/State University of Campinas (CAAE:63839222.3.0000.5418), and the Ethics Committee of the Stomatology Department of the A.C. Camargo Cancer Center (No. 3487/23). All procedures adhered to the ethical standards of the responsible institutions and national committees responsible for human experimentation, as well as the Declaration of Helsinki of 1975, revised in 2008.

Study design

The study employed a cross-sectional observational design with a sample of 215 patients diagnosed with HNC who were receiving follow-up care at OROCENTRO (Oral Lesion Diagnosis and Treatment Service) of the Piracicaba Dental School, State University of Campinas (Piracicaba, Brazil) and the Stomatology Department of the A.C. Camargo Cancer Center (São Paulo, Brazil). Patient recruitment occurred between November 2022 and November 2024. There were no specific years or date restrictions, as the selection was based on convenience, assessing all individuals who expressed willingness to participate and met the eligibility criteria.

The eligibility criteria included survivors diagnosed with head and neck squamous cell carcinoma who had completed treatment and were in follow-up for at least six months, with no evidence of disease recurrence or uncontrolled regional or distant metastases, and who were employed either part-time or full-time at the time of their cancer diagnosis. Participants were required to be 18 years or older, with no upper age limit, acknowledging that many self-employed patients in Brazil continue working beyond the typical retirement age. Only those who provided voluntary consent and signed the informed consent form were included in the study.

Data collection

All HNC survivors were followed up annually at the outpatient clinics where the research was conducted. During these routine visits, a dental surgeon trained for the research administered structured questionnaires to participants who met the eligibility criteria. The interviews were conducted before the patients' regular appointments.

Measuring instruments

The measuring instruments comprised questionnaires and scales, consisting of structured, written questions. For individuals unable to read or interpret text due to illiteracy, functional illiteracy, or visual limitations, the questionnaires were administered as interviews. All interviews were conducted by the lead researcher, who was trained to ensure consistency in data collection and to minimize the risk of influencing participants' responses.

Questionnaire on clinical information, socioeconomic level and disease data

Data were collected on demographic and socioeconomic variables, including gender (male and female), age, skin color, marital status, habits, monthly family income (categorized as: 1- Up to 1 minimum wage. 2- More than 2 to 3 minimum wages. 3- More than 5 to 10 minimum wages. 4- More than 20 minimum wages) and years of schooling (ranging from 0 to 15 years, based on 8 years of elementary school, 3 years of secondary school and 4 years of completed higher education).

Additionally, information was regathered to investigate disease characteristics, such as the type and location of cancer, tumor staging, treatments performed, medical interventions, treatment duration, and adverse effects.

These data were collected by the researcher using information previously recorded in the anamnesis, electronic medical records from the health services where the patients received care, and observations made during dental consultations.

Return to work questionnaire

Regarding RW, a structured questionnaire was applied, initially addressing the patient's profession prior to the cancer diagnosis. To assess RW after treatment, a binary question was used ("Did you RW after completing treatment?" - Yes/No), followed by additional questions depending on the response. If the answer was negative, patients were asked about the reasons for not returning. If the answer was positive, further questions explored whether the return occurred at the same workplace or a different one, and whether any adaptations or changes in work activities were necessary. A question was also included regarding the patient's perception of their ability to RW ("Do you feel unable to RW?" - Yes/No), and if yes, the reason for this perception was requested. Finally, the questionnaire included questions about the current occupational status (employed, unemployed, or retired), as well as the financial impact of cancer during and after treatment. The full content of the questionnaire is available as supplementary material (http://www.medicina.oral.com/carpeta/suppl1_27525).

Participants were classified into two occupational groups based on their job type before the diagnosis: white-collar workers and blue-collar workers. White-collar workers are associated with predominantly administrative, intellectual, or managerial roles, while blue-collar workers are engaged in manual, technical, or operational tasks that generally require practical skills and physical effort (14).

Karnofsky Performance Status Scale

The state of disability or functional impairment was assessed using the Karnofsky Performance Status Scale (KPS), an 11point measure ranging from 0% to 100%, where 0% represents death and 100% indicates normal function. Widely utilized in studies involving cancer patients, this scale serves as tool to quantify treatment outcomes, focusing on the patient's quality of life and ability to perform activities of daily living (15).

Hospital Anxiety and Depression Scale (HADS)

The Hospital Anxiety and Depression Scale (HADS) is a 14-question tool designed to evaluate anxiety and depression symptom severity. It is divided into two subscales: seven questions focus on anxiety (HADS-A), while the remaining seven address depression (HADS-D). Each question offers four response options, scored from 0 to 3, allowing for independent assessment of both subscales. In this study, the mean scores for each subscale were calculated for the respective groups (16).

University of Washington Quality of Life Scale

The University of Washington Quality of Life Questionnaire version 4 (UW-QOLv4) consists of 15 questions, including 12 specifics to disease-related aspects such as pain, appearance, activity, recreation, swallowing, chewing, speech, shoulder movement, taste, saliva production, mood, and anxiety and three general questions. Responses are rated on a scale from 0 (indicating the worst outcome) to 100 (indicating the best outcome) using a Likert-style format. For this study, a composite score ranging from 0 to 100 was derived by averaging the scores from the disease-specific questions (17).

Statistical analysis

A descriptive analysis was performed to summarize the socioeconomic, clinical, and illness-related factors, as well as employment status of the sample using frequencies and percentages. A Shapiro-Wilk test was conducted to assess the normality of continuous variables. For normally distributed variables, an independent t-test for comparisons between two independent groups was used. For non-normally distributed variables, the Mann-Whitney U test was used. Associations involving categorical variables were evaluated using Chi-square tests. A significance level of p &lt; 0.05 was considered statistically significant for all tests. The statistical analysis was performed using Python 3.11 (Python Software Foundation) and R 4.3.1 (R Foundation for Statistical Computing).

## Results

Return to work

Out of 215 participants, the majority were employed in blue-collar jobs (135; 62.8%), while the remainder held white-collar positions (80; 37.2%). Before their cancer diagnosis, most participants were in formal employment (163; 75.8%), with a smaller group self-employed (52; 24.2%). Following treatment, a significant portion managed to return to their original workplaces (137; 63.7%), highlighting their reintegration into the workforce. However, a notable group did not RW (78; 36.3%). The primary reasons for this inability included physical and functional limitations (63; 80.8%), dismissal (12; 15.4%), and psychosocial or emotional challenges (3; 3.8%).

Among those who returned to their jobs, some required workplace adaptations or role modifications (38; 27.7%), while the majority resumed work without any changes (99; 72.3%). When considering their ability to RW, most participants felt capable (149; 69.3%), whereas a smaller group believed they were unable to return (66; 30.7%). Among those who reported an inability to return, physical and functional limitations were the predominant reason (63; 95.5%), followed by psychosocial or emotional factors (3; 4.5%).

The current employment situation revealed that among the 78 participants who did not RW, outcomes varied: Half retired (39; 18.15%), primarily due to disability following cancer treatment, while the other half remained unemployed (39; 18.15%). During treatment, some participants experienced significant financial strain (56; 26.0%), whereas the majority reported no notable impact (159; 74.0%). Post-treatment, financial hardship decreased markedly, with only a minority continuing to face challenges (30; 13.9%) and most reporting no further concerns (185; 86.1%).

In terms of socioeconomic data, most participants reported a family income of up to three minimum wages (149; 69.3%), while others exceeded this threshold (66; 30.7%). Regarding education, the majority had completed up to 11 years of schooling (145; 67.4%), while a smaller group had pursued further education (70; 32.6%) (Table 1).

Table 1

Return to work and clinicopathologic aspects

The correlation tests showed that age (p=0.1446), gender (p=0.7897), marital status (p=0.2963), and skin color (p=0.1468) were also not significantly associated with RW. Tumor site was also no statistical significance (p=0.7326), although patients with oropharyngeal (77.7%) and lip (75%), showed higher RW rates. Tobacco and alcohol consumption were also not significantly associated with RW. On the other hand, clinical characteristics were more predictive. Tumor size was strongly associated with RW (p&lt;0.0001): patients with T1 (83.6%) and T2 (75.8%) tumors returned at much higher rates than those with T3 (27.5%) and T4 (42.1%). Lymph node involvement also influenced RW (p=0.0076), with N0 patients showing higher return (67.4%) compared to those with nodal metastasis. All patients with distant metastasis (M1) did not RW, while 65.0% of M0 patients did (p=0.0122).

Treatment-related variables also had a significant impact. Radical surgery did not demonstrate significant results (62.0%; p=0.4253). In contrast, patients who did not undergo radiotherapy (87.5%) or chemotherapy (73.8%) had significantly higher RW rates (p=0.0005 and p=0.0001, respectively). Similarly, patients who did not receive combined treatments such as surgery with radiotherapy (75.0%), surgery with chemotherapy (69.1%) or radiotherapy with chemotherapy (73.9%) had better outcomes. Tracheostomy was associated with significantly lower RW (12.5%; p=0.0021). Completing treatment within six months was favorable (67.5% vs. 28.6%; p=0.0004). Although not statistically significant (p=0.0991), patients with a Karnofsky score of 100% had the highest RW rate (70.9%) (Table 2).

Table 2

Return to work and side effects

The analysis of treatment side effects on RW rates revealed that neck hyperesthesia had a significant impact. Among the 30 patients with this condition, only 11 (36.7%) returned to work, whereas 126 out of 185 (68.1%) without it were able to return (p=0.0009). In contrast, patients with lip hyperesthesia had a RW rate of 18 (52.9%), compared to 119 (65.7%) of those without this condition (p=0.1542). Similarly, chin hyperesthesia showed a RW rate of 12 (48.0%), compared to 125 (65.8%) of those unaffected (p=0.0820). Cheek hyperesthesia also showed a RW rate of 12 (48.0%), compared to 125 (65.8%) of those without it (p=0.0820). For tongue hyperesthesia, 23 (52.3%) patients returned to work compared to 114 (66.7%) of those without the condition (p=0.0902). For paresthesia, none of the 5 (100%) patients with incomplete eye closure returned to work, while 137 (65.2%) without this condition did (p=0.0027). Patients with a suspended corner of the mouth had a significantly lower RW rate of three (27.3%), compared to 134 (65.7%) of those without it (p=0.0098). For inability to wrinkle the forehead, two (33.3%) returned to work compared to 135 (64.6%) without the condition (p=0.1164). Similarly, patients with lack of lip seal had a RW rate of six (42.9%), compared to 131 (65.2%) without it (p=0.0931).

Among functional restrictions, patients with speech restriction showed a significantly reduced RW rate of 52 (51.0%), compared to 85 (75.2%) of those without it (p=0.0002). Taste restriction also significantly reduced RW rates, with 34 (39.1%) of affected patients returning to work compared to 103 (80.5%) of unaffected patients (p&lt;0.0001). Swallowing restriction resulted in 44 (44.0%) returning to work compared to 93 (80.9%) without the restriction (p&lt;0.0001). For breathing restriction, only 15 (30.6%) returned to work compared to 122 (73.5%) without the restriction (p&lt;0.0001). Mobility restriction had the strongest negative impact, with just three (15.8%) patients returning to work compared to 134 (68.4%) of those without it (p&lt;0.0001). Salivation restriction was also significant, as 83 (55.7%) returned to work compared to 54 (81.8%) without it (p=0.0002). For treatment-related conditions, radiation-induced caries did not significantly affect RW rates, with six (60.0%) of affected patients returning to work compared to 131 (63.9%) without it (p=0.8021). Similarly, osteoradionecrosis was not significant, with a RW rate of 18 (66.6%) for affected patients compared to 119 (63.3%) of those without the condition (p=0.7335) (Table 3).

Table 3

Association between return to work, workplace aspects and socioeconomic level

The analysis for job category showed that white-collar workers had a higher RW rate of 60 (75.0%), compared to blue-collar workers, who had a RW rate of 77 (57.0%) (p=0.0081). Regarding employment status before cancer diagnosis, employed patients had a RW rate of 95 (58.3%), while self-employed patients had a higher rate of 42 (80.7%) (p=0.0033). For reasons for not returning to work, all patients dismissed (12; 100%) and those citing physical and functional limitations (63; 100%) or psychosocial and emotional factors (3; 100%) (p=1.0). Patients who returned to the same workplace all reported a RW rate of 137 (100.0%), while none of the patients who did not return to the same workplace resumed work (0; 0%) (p&lt;0.0001). For adaptation or change of function necessary to RW, patients who required adaptations all returned to work (38; 100.0%) (p=1.0). Similarly, patients not requiring changes also had a RW rate of 99 (100.0%), while none of those who did not RW underwent adaptations (0%).

Regarding the perception of work ability, only one patient (1.5%) among the 137 who returned to work reported feeling unable to do so, while the vast majority (136; 91.3%) perceived themselves as able to return (p&lt;0.0001). Among those who considered themselves unable to RW, 63 (100%) attributed their inability to physical and functional limitations. Additionally, 3 patients reported psychosocial and emotional factors as barriers: 2 (66.7%) did not RW, and 1 (33.3%) returned despite perceiving such difficulties (p=0.0844). Regarding current employment status, all the patients who returned to work remained employed (137; 100%). Among the unemployed, 39 remained unemployed, and another 39 patients had retired (p&lt;0.0001).

For financial impact on life and/or family during cancer treatment, only 13 (23.2%) of patients who reported financial impact returned to work, compared to 124 (96.7%) of those who did not report an impact (p&lt;0.0001). A similar trend was observed for financial impact after cancer treatment, with only one (3.3%) of those reporting financial impact returning to work, compared to 136 (73.5%) of those without financial impact (p&lt;0.0001). Regarding socioeconomic level, patients with a household income of 3 had a RW rate of 85 (57.0%), while those with &gt;3 had a rate of 52 (78.8%) (p=0.0022). For highest grade completed in school, patients with 11 years of education had a RW rate of 84 (57.9%), compared to 53 (75.7%) for those with &gt;11 years of education (p=0.0110) (Table 4).

Table 4

Association between return to work, psychological, and quality of life measures

Patients who returned to work showed significantly higher levels of anxiety, as assessed by the HADS-A scale, with 69.3% achieving scores higher than 1.55, compared to 55.1% of those who did not return (p=0.0366). Regarding depression, although no statistically significant difference was observed in HADS-D scores (p=0.1079), a trend toward lower depressive symptoms was noted among those who returned to work: 66.7% had scores above 1.74, compared to 44.5% of those who did not return. In addition, patients who returned to work showed better quality of life results according to the UW-QOLv4 scale. Specifically, 89.1% of those who returned to work recorded scores above 32.19, while only 21.8% of those who did not return reached this level (p&lt;0.0001) (http://www.medicina.oral.com/carpeta/suppl2_27525).

## Discussion

RW has become an essential aspect of rehabilitation for HNC survivors, contributing to their personal development, self-esteem, and overall quality of life (1 , 6 , 18). This study focused on individuals who had completed treatment at least six months prior, aiming to address the limited research in Brazil on factors influencing RW in this population. By examining the relationships between RW, clinicopathological and socioeconomic characteristics, and treatment side effects, this research provides a better understanding of the challenges faced by HNC survivors in the country.

The analysis of social and occupational reintegration revealed that most patients held blue-collar jobs involving considerable physical effort prior to diagnosis. The observed RW rate was consistent with findings from other studies (19 - 21). Among those who returned to work, some required role adaptations, although the majority resumed their activities without changes. These findings highlight the challenges faced by survivors, as treatment related limitations often necessitate workplace adjustments, similarly to what was reported by Agarwal et al. (19), who found that 24.1% of survivors experienced changes in job functions or roles due to health-related constraints. These results highlight the enduring challenges faced by survivors, as treatment and health-related constraints often necessitate adjustments to their professional lives. A systematic review further emphasizes that, although many HNC survivors achieve RW, a considerable number remain unable to work or must reduce their hours, illustrating the lasting impact of HNC on survivors' quality of life and daily activities (18).

The reasons for not RW among HNC survivors were categorized into dismissal, physical and functional limitations, and psychosocial and emotional factors. In this study, 36.3% of survivors did not RW, consistent with previous research (18 - 20). Physical and functional limitations, including oral dysfunctions, dry mouth, changes in appearance, fatigue, pain, and weakness, were the most significant barriers. Additionally, 12 patients cited redundancy as the reason for not returning, reflecting studies showing HNC survivors face higher risks of dismissal and unemployment compared to other cancer survivors (1). Psychosocial and emotional factors also contributed, highlighting the need for tangible support to enhance motivation for RW (20). Furthermore, 30.7% of survivors considered themselves unable to return, underscoring the importance of interventions. A review by Zecena Morales et al (6)., emphasized physical and functional limitations as primary challenges to RW, emphasizing the need for tailored strategies to improve these challenges.

This study revealed that none of the 39 patients who retired after treatment had considered retirement prior to their diagnosis, and 39 of survivors were unemployed. These findings underscore the critical need for public policies that provide economic support and opportunities for professional requalification, particularly during periods of heightened social and economic vulnerability. Cancer's impact on employment is profound, often resulting in increased unemployment and financial strain (6). In this study, most survivors faced socioeconomic vulnerability, characterized by limited education and low household income. Although the proportion of individuals reporting financial difficulties decreased after treatment, many continued to experience economic hardship due to their inability to RW, which contributes to long-term income loss and persistent challenges for their families (22).

Regarding RW and clinicopathological features in HNC survivors, no statistically significant associations were found for age, gender, marital status, skin color, smoking, or alcohol consumption. However, survivors who returned to work were younger on average, consistent with prior research that identifies age as an important factor facilitating work reintegration (1). Gender differences were minimal, with men and women showing similar rates of RW, although some studies, like Agarwal et al (19)., reported higher RW rates for women, while Cleck et al (23)., found women more prone to work discontinuity.

Marital status also did not show a significant correlation in our analysis, despite evidence from other studies suggesting that married individuals are more likely to RW, while those living alone face higher unemployment risks (6 , 19 , 20). Similarly, skin color, smoking, and alcohol consumption showed no significant influence, although prior research links smoking and alcohol use to lower RW rates (1 , 13 , 23). These findings highlight the diverse factors influencing RW and emphasize the variability in data that impact RW outcomes.

The tumor location, while showing some trends, was not significantly associated with RW rates in this study. However, patients with oropharyngeal and lip tumors demonstrated higher RW rates. Previous research has analyzed tumor location by specific subgroups (6 , 20 , 21 , 23 , 24), with studies on oropharyngeal tumors reporting particularly high RW rates among patients with HPV-positive oropharyngeal cancer (24). Additionally, one study found that patients with oral cancer were about five times more likely to RW compared to those with tumors in other head and neck regions (25).

Tumor stage emerged as a key factor influencing RW, with early-stage tumors favoring occupational reintegration. This likely reflects the less intensive treatments and milder side effects experienced by these patients, enabling a smoother RW. Conversely, advanced stages require more aggressive multimodal therapies and often cause greater functional impairment, significantly hampering survivors' ability to resume their professional activities. These findings underscore the profound impact of tumor stage on workplace reintegration (5 , 6 , 10 , 13).

Regarding treatment type, surgery alone did not significantly affect RW rates but was associated with more favorable outcomes compared to multimodal approaches, such as surgery combined with chemotherapy or radiotherapy, which often result in greater impairments (7 , 20 , 25 , 26). Patients who did not receive radiotherapy or chemotherapy demonstrated higher RW rates, consistent with Taylor et al. (27), who reported increased disability linked to chemotherapy in HNC survivors. The findings suggest that combined treatments may amplify side effects, reduce quality of life, and hinder workforce reintegration. Moreover, multimodal therapies are frequently employed in more advanced disease stages (2), further complicating RW and adding barriers to recovery.

The use of tracheostomy was found to negatively impact RW, consistent with previous studies (28). Shorter treatment durations were associated with better RW outcomes, whereas longer treatments had a detrimental effect, as highlighted in earlier research (20). Persistent side effects of HNC treatments, commonly referred to as treatment toxicities, significantly limit survivors' ability to perform daily activities and RW (29). These effects not only reduce work capacity but also adversely affect the overall RW experience, often requiring workplace adjustments (6).

This study uniquely examined the role of sensory alterations, specifically hyperesthesia and paresthesia, in RW, with neck hyperesthesia having the most pronounced negative impact. No prior research has addressed these aspects in detail. Our findings indicate that sensory disturbances including neck hyperesthesia, paresthesia with incomplete eyelid closure, and paresthesia at the corner of the mouth significantly impair RW. Functional challenges such as breathing restrictions, mobility limitations, speech and swallowing difficulties, and taste alterations were also strongly associated with reduced RW. These results align with Chen et al. (20) who reported that survivors failing to RW experienced a greater symptom burden than those who successfully reintegrated into the workforce.

Many studies do not explore survivors occupations in depth, making it challenging to fully understand how work affects their return. In this study, survivors were classified as white-collar or blue-collar workers, revealing significant occupational disparities. White-collar workers were more likely to RW, while blue-collar workers faced greater obstacles, often due to the physical demands of their roles. This aligns with previous research (1 , 6 , 19 , 23 , 24). The analysis also showed that self-employed survivors had a higher likelihood of returning to work compared to employees, supporting findings by Miller et al (26)., who noted that self-employed individuals were twice as likely to return. Their financial responsibility and workplace flexibility, including reduced hours or adjusted roles, likely contribute to this higher rate. Financial difficulties emerged as a major barrier to RW, highlighting the need for policies that alleviate the financial burdens faced by survivors, as emphasized in a study from Taiwan (20). Furthermore, workplace adaptations, like functional adjustments, were found to significantly assist survivors in successfully returning to work (10).

Finally, in the present study, it was observed that survivors who returned to work had higher levels of anxiety, which may be associated with a combination of factors specific to the process of job reintegration. In our sample, as already mentioned, the patients who most returned to work were predominantly professionals in white-collar occupations, whose activities are associated with intellectual, administrative and technical functions (24). These professions can exert greater pressure on survivors, since the expectation to perform well, especially after a period of absence from work, can lead to doubts about one's ability. On the other hand, previous studies have indicated that survivors who returned to work had lower levels of anxiety and depressive symptoms (10 , 18 , 24 , 25 , 28). In our study, patients who returned to work had lower rates of depression compared to those who did not, consistent with findings from other studies (10 , 18 , 24 , 25 , 28). In addition, a better quality of life was often observed among survivors who returned to work (22 , 25 , 26 , 30) which is in line with the results obtained in our study.

## Conclusions

The results of this study highlight the multidimensionality of returning to work among HNC survivors, revealing an interaction between clinical, socioeconomic, and occupational factors. The prevalence of RW was considerably significant (63.7%), but barriers related to educational level, tumor size, presence of metastasis, type of treatment, and its side effects challenged it. White-collar workers had higher rates of RW, indicating that the nature of the job influences the ability to reintegrate into work. In addition, psychosocial aspects, such as high levels of anxiety in survivors who returned to work, may be related to the emotional and psychological demands of the recovery process after a period of absence. To support these survivors, it is essential to adopt personalized multidisciplinary strategies to address physical and functional limitations and socioeconomic inequalities, as well as mental health, thus improving the reintegration and quality of life of survivors of HNC.

## Figures and Tables

**Table 1:** Table Data of the patients about the return to work and socioeconomic level.

Return to Work			Total (%)
Job Category		White-collar	80 (37.2%)
		Blue-collar	135 (62.8%)
Employment status before cancer diagnosis		Employed	163 (75.8%)
		Self-employed	52 (24.2%)
Return to work after completing cancer treatment		Yes	137 (63.7%)
		No	78 (36.3%)
The reasons for not returning to work		Dismissed	12 (15.4%)
		Physical and Functional Limitations	63 (80.8%)
		Psychosocial and Emotional Factors	3 (3.8%)
Return to the same workplace		Yes	137 (63.7%)
		No	78 (36.3%)
Adaptation or change of function necessary to return to work		Yes	38 (27.7%)
		No	99 (72.3%)
Consider yourself unable to return to work		Yes	66 (30.7%)
		No	149 (69.3%)
If yes, for what reason do you consider yourself unable to return to work?		Physical and Functional Limitations	63 (95.5%)
		Psychosocial and Emotional Factors	3 (4.5%)
Current employment status		Employed	137 (63.7%)
		Unemployed	39 (18.15%)
		Retired	39 (18.15%)
Financial impact on your life and/or your family during cancer treatment		Yes	56 (26.0%)
		No	159 (74.0%)
Financial impact after cancer treatment		Yes	30 (13.9%)
		No	185 (86.1%)
Socioeconomic level	Last month, how much income, in reais, did all the people living in your household receive, considering salaries, social benefits (e.g., Bolsa FamÃ­lia), pension, rent, retirement, or other incomes? (Mean: 3; Range: 1-4)	≤ 3	149 (69.3%)
		> 3	66 (30.7%)
	What is the highest grade you completed in school? (Mean: 11; Range: 8-15)	≤ 11	145 (67.4%)
		> 11	70 (32.6%)

1

**Table 2:** Table Relation between return to work and clinicopathologic features.

Clinicopathologic features			Return to work		
			Yes (%)	No (%)	p-value
Age (mean: 58.35 years)		≤ 58.35	65 (69.1%)	29 (30.9%)	0.1446
		> 58.35	72 (59.5%)	49 (40.5%)	
Sex		Male	96 (63.2%)	56 (36.8%)	0.7897
		Female	41 (65.1%)	22 (34.9%)	
Marital status		Single	11 (68.8%)	5 (31.2%)	0.2963
		Married	120 (63.2%)	70 (36.8%)	
		Divorced	4 (100%)	0 (0%)	
		Widowed	2 (40%)	3 (60%)	
Skin color		White	101 (67.8%)	48 (32.2%)	0.1468
		Mixed-race	12 (60.0%)	8 (40.0%)	
		Black	24 (52.2%)	22 (47.8%)	
Site		Floor of the mouth	11 (55.0%)	9 (45.0%)	0.7326
		Gingiva	2 (50.0%)	2 (50.0%)	
		Hard palate	7 (63.6%)	4 (36.4%)	
		Lip	12 (75.0%)	4 (25.0%)	
		Oropharynx	14 (77.7%)	4 (22.3%)	
		Soft palate	7 (70.0%)	3 (30.0%)	
		Tongue	45 (70.3%)	19 (29.7%)	
		Others	39 (54.2%)	33 (45.8%)	
Smoking		Yes	95 (62.1%)	58 (37.9%)	0.4350
		No	42 (67.7%)	20 (32.3%)	
Alcoholism		Yes	73 (64.0%)	41 (36.0%)	0.9189
		No	64 (63.3%)	37 (36.4%)	
Tumor size (T)		T1	51 (83.6%)	10 (16.4%)	< 0.0001
		T2	50 (75.8%)	16 (24.2%)	
		T3	14 (27.5%)	37 (72.5%)	
		T4	8 (42.1%)	11 (57.9%)	
		Tx	0 (0%)	1 (100%)	
		NR	14 (82.3%)	3 (17.7%)	
Lymph node metastasis (N)		N0	93 (67.4%)	45 (32.6%)	0.0076
		N1	14 (44.1%)	19 (55.9%)	
		N2	7 (46.6%)	8 (53.3%)	
		N3	1 (12.5%)	7 (87.5%)	
		Nx	3 (100%)	0 (0%)	
		NR	14 (82.4%)	3 (17.6%)	
Distant metastasis (M)		M0	115 (65.0%)	62 (35.0%)	0.0122
		M1	0 (0%)	4 (100%)	
		Mx	8 (47.0%)	9 (53.0%)	
		NR	14 (82.3%)	3 (17.7%)	
Treatment	Radical surgery	Yes	93 (62.0%)	57 (38.0%)	0.4253
		No	44 (67.7%)	21 (32.3%)	
	Radiotherapy	Yes	102 (58.3%)	73 (41.7%)	0.0005
		No	35 (87.5%)	5 (12.5%)	
	Chemotherapy	Yes	41 (48.2%)	44 (51.8%)	0.0001
		No	96 (73.8%)	34 (26.2%)	
	Radical surgery + Radiotherapy	Yes	59 (53.2%)	52 (46.8%)	0.0009
		No	78 (75.0%)	26 (25.0%)	
	Radical surgery + Chemotherapy	Yes	16 (40.0%)	24 (60.0%)	0.0005
		No	121 (69.1%)	54 (30.9%)	
	Radiotherapy + Chemotherapy	Yes	41 (48.2%)	44 (51.8%)	0.0001
		No	96 (73.9%)	34 (26.1%)	
	Tracheostomy	Yes	1 (12.5%)	7 (87.5%)	0.0021
		No	136 (65.7%)	71 (34.3%)	
Time treatment		≤ 6 months	131 (67.5%)	63 (32.5%)	0.0004
		> 6 months	6 (28.6%)	15 (71.4%)	
Karnofsky Scale (level)		100%	73 (70.9%)	30 (29.1%)	0.0991
		90%	38 (57.5%)	28 (42.5%)	
		80%	25 (59.5%)	17 (40.5%)	
		70%	1 (25.0%)	3 (75.0%)	

2

**Table 3:** Table Relation between return to work and side effects.

Treatment side effects				Return to work		
				Yes (%)	No (%)	p-value
Sensory Impairments	Hyperesthesia	Neck	Yes	11 (36.7%)	19 (63.3%)	0.0009
			No	126 (68.1%)	59 (31.9%)	
		Lip	Yes	18 (52.9%)	16 (47.1%)	0.1542
			No	119 (65.7%)	62 (34.3%)	
		Chin	Yes	12 (48.0%)	13 (52.0%)	0.0820
			No	125 (65.8%)	65 (34.2%)	
		Cheek	Yes	12 (48.0%)	13 (52.0%)	0.0820
			No	125 (65.8%)	65 (34.2%)	
		Tongue	Yes	23 (52.3%)	21 (47.7%)	0.0902
			No	114 (66.7%)	57 (33.3%)	
	Paresthesia	Incomplete eye closure	Yes	0 (0%)	5 (100.0%)	0.0027
			No	137 (65.2%)	73 (34.8%)	
		Suspended corner of the mouth	Yes	3 (27.3%)	8 (72.7%)	0.0098
			No	134 (65.7%)	70 (34.3%)	
		Inability to wrinkle the forehead	Yes	2 (33.3%)	4 (66.6%)	0.1164
			No	135 (64.6%)	74 (35.4%)	
		Lack of lip seal	Yes	6 (42.9%)	8 (57.1%)	0.0931
			No	131 (65.2%)	70 (34.8%)	
Functional Restrictions		Speech restriction	Yes	52 (51.0%)	50 (49.0%)	0.0002
			No	85 (75.2%)	28 (24.8%)	
		Taste restriction	Yes	34 (39.1%)	53 (60.9%)	< 0.0001
			No	103 (80.5%)	25 (19.5%)	
		Swallowing restriction	Yes	44 (44.0%)	56 (56.0%)	< 0.0001
			No	93 (80.9%)	22 (19.1%)	
		Breathing restriction	Yes	15 (30.6%)	34 (69.4%)	< 0.0001
			No	122 (73.5%)	44 (26.5%)	
		Mobility restriction	Yes	3 (15.8%)	16 (84.2%)	< 0.0001
			No	134 (68.4%)	62 (31.6%)	
		Salivation restriction	Yes	83 (55.7%)	66 (44.3%)	0.0002
			No	54 (81.8%)	12 (18.2%)	
Treatment-Related Conditions		Radiation-induced caries	Yes	6 (60.0%)	4 (40.0%)	0.8021
			No	131 (63.9%)	74 (36.1%)	
		Osteoradionecrosis	Yes	18 (66.6%)	9 (33.3%)	0.7335
			No	119 (63.3%)	69 (36.7%)	

3

**Table 4:** Table Relation between return to work, workplace aspects and socioeconomic level.

Workplace aspects			Return to work		
			Yes (%)	No (%)	P-value
Job Category		White-collar	60 (75.0%)	20 (25.0%)	0.0081
		Blue-collar	77 (57.0%)	58 (43.0%)	
Employment status before cancer diagnosis		Employed	95 (58.3%)	68 (41.7%)	0.0033
		Self-employed	42 (80.7%)	10 (19.3%)	
The reasons for not returning to work		Dismissed	0 (0%)	12 (100.0%)	1.0
		Physical and Functional Limitations	0 (0%)	63 (100.0%)	
		Psychosocial and Emotional Factors	0 (0%)	3 (100.0%)	
Return to the same workplace		Yes	137 (100.0%)	0 (0%)	< 0.0001
		No	0 (0%)	78 (100%)	
Adaptation or change of function necessary to return to work		Yes	38 (100.0%)	0 (0%)	1.0
		No	99 (100.0%)	0 (0%)	
Consider yourself unable to return to work		Yes	1 (1.5%)	65 (98.5%)	< 0.0001
		No	136 (91.3%)	13 (8.7%)	
If yes, for what reason do you consider yourself unable to return to work?		Physical and Functional Limitations	0 (0%)	63 (100%)	0.0844
		Psychosocial and Emotional Factors	1 (33.3%)	2 (66.7%)	
Current employment status		Employed	137 (100%)	0 (0 %)	< 0.0001
		Unemployed	0 (0%)	39 (100%)	
		Retired	0 (0%)	39 (100%)	
Financial impact on your life and/or your family during cancer treatment		Yes	13 (23.2%)	43 (76.8%)	< 0.0001
		No	124 (96.7%)	35 (3.3%)	
Financial impact after cancer treatment		Yes	1 (3.3%)	29 (96.6%)	< 0.0001
		No	136 (73.5%)	49 (26.5%)	
Socioeconomic level	Last month, how much income, in reais, did all the people living in your household receive, considering salaries, social benefits (e.g., Bolsa FamÃ­lia), pension, rent, retirement, or other incomes? (Mean: 3; Range: 1-4)	≤ 3	85 (57.0%)	64 (43.0%)	0.0022
		> 3	52 (78.8%)	14 (21.2%)	
	What is the highest grade you completed in school? (Mean: 11; Range: 8-15)	≤ 11	84 (57.9%)	61 (42.1%)	0.0110
		> 11	53 (75.7%)	17 (24.3%)	

4

## Data Availability

Declared none.
